# Antimicrobial Susceptibility and Antibacterial Mechanism of Limonene against *Listeria monocytogenes*

**DOI:** 10.3390/molecules25010033

**Published:** 2019-12-20

**Authors:** Yingjie Han, Zhichang Sun, Wenxue Chen

**Affiliations:** College of Food Sciences & Engineering, Hainan University, 58 People Road, Haikou 570228, China; 18085231210009@hainanu.edu.cn

**Keywords:** Limonene, antibacterial mechanism, membrane damage, nucleic acid, protein, ATP, ATPase, respiratory complexes

## Abstract

Limonene is a monoterpenoid compound, which is founded in a lot of plants’ essential oils with good antibacterial activity against food-borne pathogens, but it has an ambiguous antimicrobial susceptibility and mechanism against Listeria monocytogenes *(L. monocytogenes)*. In this study, the antimicrobial susceptibility of Limonene to *L. monocytogenes* was studied, and some new sights regarding its antibacterial mechanism were further explored. Scanning electron microscopy (SEM) verified that limonene caused the destruction of the cell integrity and wall structure of *L. monocytogenes.* The increase in conductivity and the leakage of intracellular biomacromolecules (nucleic acids and proteins) confirmed that limonene had an obvious effect on cell membrane permeability. The results of Propidium Iodide (PI) fluorescence staining were consistent with the results of the conductivity measurements. This indicated that limonene treatment caused damage to the *L. monocytogenes* cell membrane. Furthermore, the decrease in ATP content, ATPase (Na^+^K^+^-ATPase, Ca^2+^-ATPase) activity and respiratory chain complex activity indicated that limonene could hinder ATP synthesis by inhibiting the activity of the respiratory complex and ATPase. Finally, differential expression of proteins in the respiratory chain confirmed that limonene affected respiration and energy metabolism by inhibiting the function of the respiratory chain complex.

## 1. Introduction

Food-borne pathogens are one of the main causes of foodborne diseases and they have become an important public health problem threatening people’s health [1]. The Gram-positive bacterium *L. monocytogenes* is widely distributed in a variety of foods (meat, aquatic products, dairy products, vegetables, etc.) [2] and it is one of the most common zoonotic foodborne pathogens that can invade the body by food chain, causing meningitis, myocarditis, sepsis, premature birth, and other diseases in humans and livestock [3]. In addition, *L. monocytogenes* has a strong adaptability to environments. For example, it can survive at cold storage temperatures, in low pH values and in high salt concentrations [4]. Therefore, controlling the *L. monocytogenes* pollution is a major issue in the face of food safety [5]. In response to *L. monocytogenes* contamination, a variety of different antimicrobial agents have been used in recent years. Among them, natural antimicrobial agents have become the focus [6].

Essential oils are considered to be potential natural food preservatives and antimicrobial agents and have been widely used in food preservation [7]. It was reported that terpenoids were an important component of essential oils [8]. Limonene (1-methyl-4-(1-methylethenyl) is one of the most common terpenes in nature and is widely found in the volatile oils of various plants (black pepper, lemon and orange, etc.) [9]. Limonene has broad application prospects in antibacterial and food preservation due to its broad-spectrum bactericidal activity, safety, and low toxicity [10]. Moreover, D-limonene can significantly inhibit gram-negative and gram-positive bacteria as well as fungal activity [11]. In addition, many researchers have confirmed that D-limonene can effectively inhibit the growth of spoilage bacteria, such as *Aspergillus niger*, *Pseudomonas aeruginosa, Staphylococcus aureus*, and *Escherichia coli* [12,13]. Limonene, which is the main ingredient of lemon essential oil, was found to have antimicrobial activities against *L. monocytogenes* in minced beef meat [14]. However, few studies have investigated the antibacterial mechanism of limonene.

The aims of this study were to determine the antibacterial susceptibility and its antibacterial mechanism of limonene against *L. monocytogenes*. The minimum inhibitory concentration (MIC) was used to evaluate the anti-*L. monocytogenes* susceptibility of limonene. The growth curves of the bacterial were determined for evaluating the effect of limonene on the growth and reproduction of *L. monocytogenes*. The mechanism of action of limonene was explored by analysing its influence on the cell morphology, membrane permeability and changes in the protein, nucleic acid, ATP, ATPase (Na^+^K^+^-ATPase, Ca^2+^-ATPase), respiratory chain complex I~V, and differential protein expression of the respiratory chain complex of *L. monocytogenes.*

## 2. Results

### 2.1. Antibacterial Susceptibility and Determination of the Growth Curves of *L. monocytogenes*

#### 2.1.1. Determination of Minimum Inhibitory Concentration (MIC)

As shown in Table 1, sterile water and 20% ethanol had no effect on bacterial growth. Limonene and positive control (Levofloxacin Hydrochloride) can both significantly inhibit bacterial reproduction, and antimicrobial abilities increased with increasing drug concentration. In addition, bacteria did not grow when the concentration of limonene was 20 mL/L. Therefore, the MIC of limonene was 20 mL/L. Meanwhile, the bacteria were inhibited by 0.625 mL/L of Levofloxacin Hydrochloride. Therefore, the MIC of Levofloxacin Hydrochloride was 0.625 mL/L. In general, limonene was less susceptible than Levofloxacin Hydrochloride against *L. monocytogenes*.

#### 2.1.2. Bacterial Growth Curves of *L. monocytogenes*

**Figure 1:**
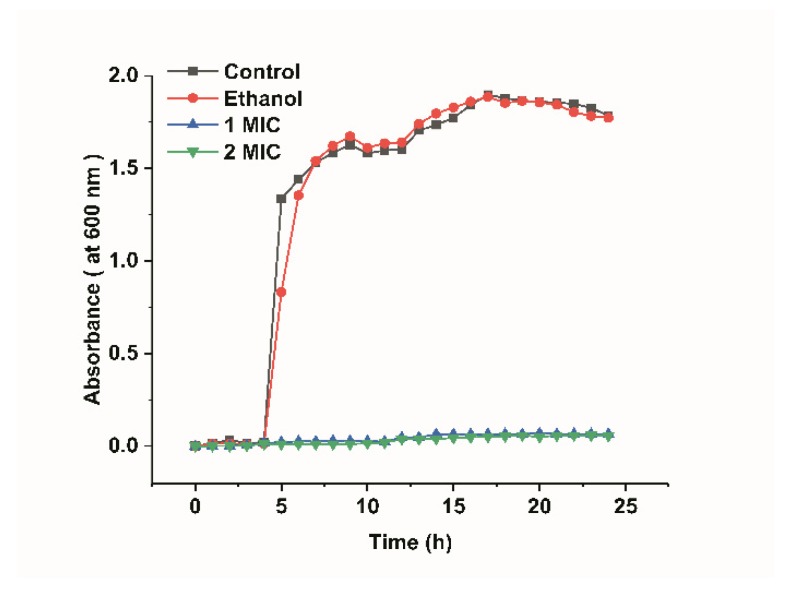
Growth curves of *L. monocytogenes.*

Figure 1 shows the growth curves of *L. monocytogenes* and they were determined by measuring the optical density at 600 nm. The growth curves of *L. monocytogenes* included four phases: lag phase (the preparatory phase for the beginning of a split), logarithmic phase (a phase of logarithmic increase in the number of bacteria), stationary phase (a phase of equilibrium in bacterial concentration), and decline phase (a phase in which the total viable count decreased significantly). As shown in Figure 1, the growth curves of the *L. monocytogenes* in the control groups showed an “S” trend. The *L. monocytogenes* of the blank control group and ethanol group both reached the logarithmic phase after 4 h, reaching their maximum at 17 h. In addition, they almost simultaneously reached the stationary phase, which indicated that 20% ethanol had no significant effect on bacterial growth (*p* > 0.05). However, the OD600 of the bacterial fluid that was treated with limonene (1 MIC and 2 MIC) was critically lower than that of the control groups, and the growth of *L. monocytogenes* almost stopped. The minimum limonene concentration that can inhibit bacterial growth and reproduction is defined as 1 MIC. Double the minimum concentration of limonene that inhibits bacterial growth and reproduction is defined as 2 MIC.

### 2.2. Antibacterial Mechanism

#### 2.2.1. Effect of Limonene on the Cell Morphology of *L. monocytogenes*

**Figure 2:**
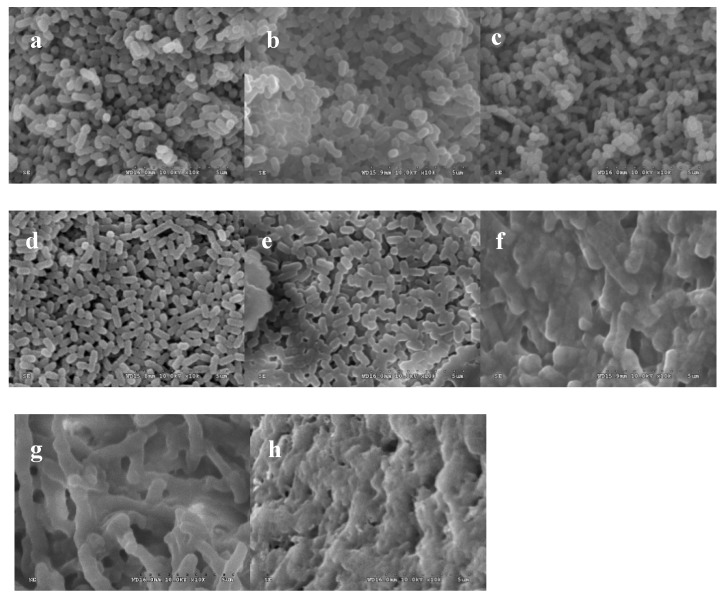
Scanning electron microphotographs of *L. monocytogenes*. Cells without treatment for 6 h (**a**), cells without treatment for 12 h (**b**), cells treated with ethanol for 6 h (**c**), cells treated with ethanol for 12 h (**d**), cells treated with limonene (1 MIC) for 6 h (**e**), cells treated with limonene (1 MIC) for 12 h (**f**), cells treated with limonene (2 MIC) for 6 h (**g**), and cells treated with limonene (2 MIC) for 12 h (**h**).

The cells in the blank control group and negative control group were intact and smooth, and no cell damage occurred, as shown in Figure 2, t (Figure 2a–d). In contrast, bacterial cells that were treated with limonene at 1 MIC and 2 MIC for 6 h and 12 h at 37 °C were subjected to considerable damage (Figure 2e,f). The cells that were exposed to limonene (1 MIC) for 6 h showed a dramatic morphological change, with alterations in shape and unclear cell boundaries (Figure 2e). As the treatment time was prolonged to 12 h, the cells showed severe ruptures and holes and severe aggregation and overlap (Figure 2f). Moreover, the cells that were treated with limonene at 2 MIC were more badly hurt than those that were treated with limonene at 1 MIC for 6 h (Figure 2g) and even completely dissolved at 12 h (Figure 2h). 

#### 2.2.2. Effect of Limonene on Cell Membrane Permeability of *L. monocytogenes*

**Figure 3:**
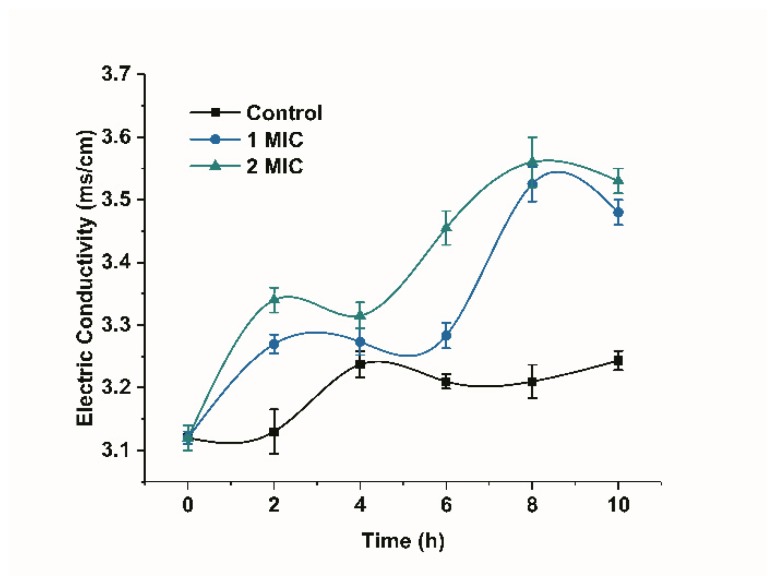
The inhibitory effects of limonene on the membrane conductivity of *L. monocytogenes.*

Changes in the conductivity of the bacterial suspension can reflect changes in the permeability of the cell membrane [15]. The conductivity of the bacterial suspension in the control group increased, but the overall change was not significant, as shown in Figure 3. However, the conductivity showed a significant increasing trend in the presence of limonene (1 MIC and 2 MIC) for a period of time (*p* < 0.05) when compared with the control group. The increase in the membrane conductivity of the bacteria treated with limonene at 2 MIC was higher than that treated with limonene at 1 MIC.

#### 2.2.3. Lethal Effect of Limonene on *L. monocytogenes*

**Figure 4:**
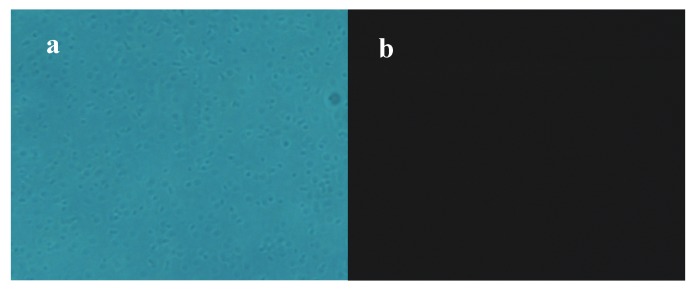

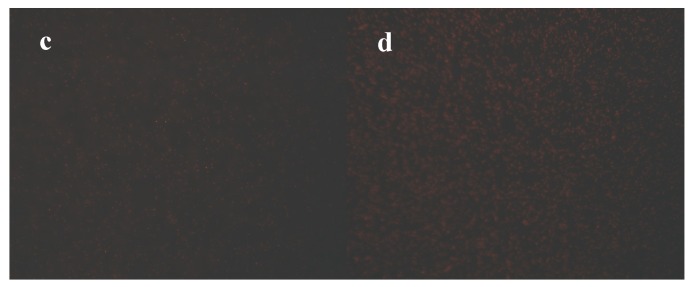
Observation of *L. monocytogenes* by using fluorescence microscope. *L. monocytogenes* under optical microscope (**a**), *L. monocytogenes* under fluorescence microscope (**b**), *L. monocytogenes* treated with limonene (MIC) for 6 h (**c**), *L. monocytogenes* treated with limonene (2 MIC) for 6 h (**d**).

PI can enter the cell membrane of dead cells, bind to nucleic acids, and emit red fluorescence but cannot pass through the membrane of living cells [16]. Therefore, the degree of PI staining can reflect the degree of cell membrane damage and the degree of cell death. The optical microscopy images (Figure 4a) showed that most bacteria in the control were growing and alive, and fluorescence images (Figure 4b) indicated that no fluorescence was detected in the control group, which further confirmed that there was almost no apoptosis of bacteria. In contrast, a small amount of red fluorescence was observed in cells that were treated with limonene at 1 MIC (Figure 4c). Moreover, the red fluorescence intensities of bacteria treated with limonene at 2 MIC were significantly enhanced (Figure 4d). 

#### 2.2.4. Nucleic Acid Leakage of *L. monocytogenes*

**Figure 5:**
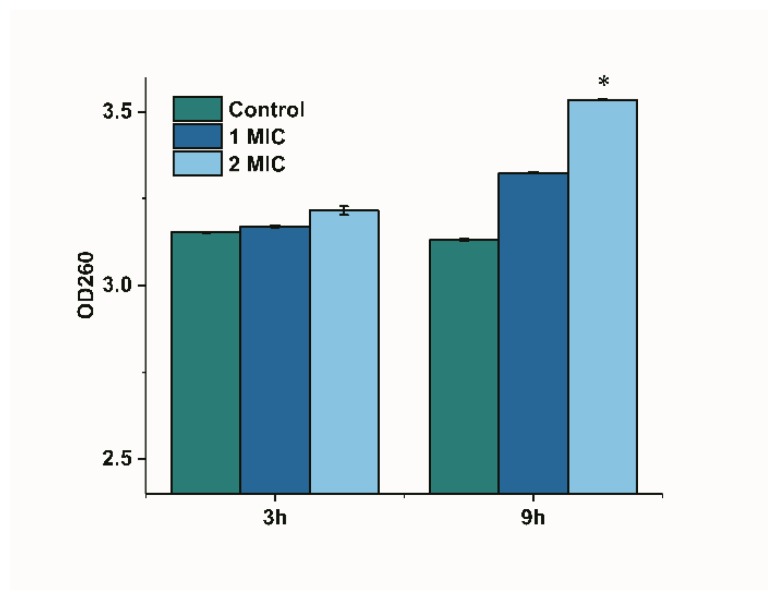
Changes of OD 260 of *L. monocytogenes.*

The release of cytoplasmic components can be monitored if the bacterial membrane is damaged [17]. We monitored the change in the optical density of limonene-treated bacterial suspensions at 260 nm to reflect the leakage of nucleic acids since nucleic acids have a strong UV absorption at 260 nm. The OD 260 in the control group showed no significant difference, as shown in Figure 5. The OD260 of the culture treated with limonene (1 MIC) for 9 h was higher than that of 3 h, but showed no significant difference. The OD 260 significantly increased (*p* < 0.05) at 9 h as compared with 3 h after treating with limonene (2 MIC).

#### 2.2.5. Effect of Limonene on the Proteins of *L. monocytogenes*

**Figure 6:**
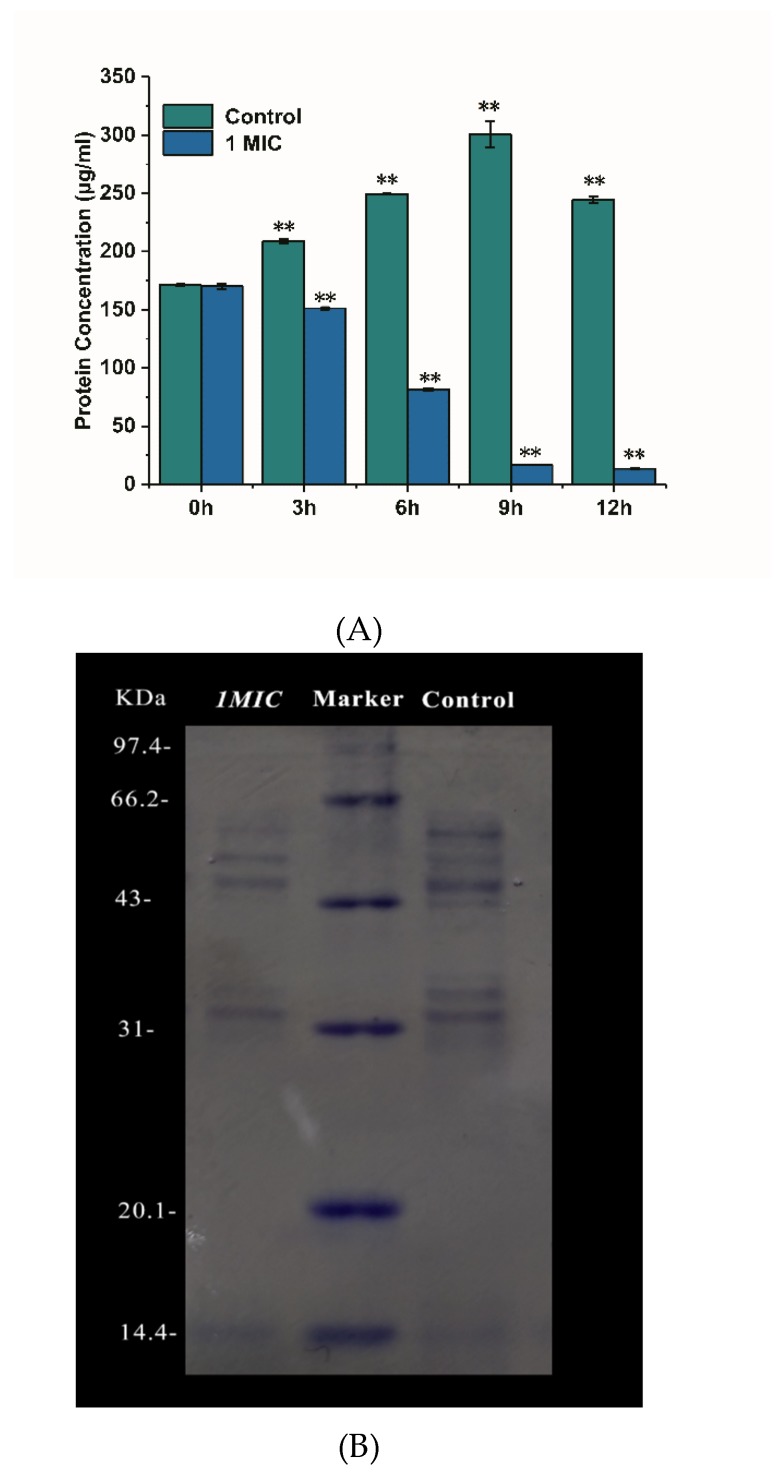
Effect of limonene on protein concentration of *L. monocytogenes* (**A**). The gel electrophoresis image of intracellular protein in *L. monocytogenes* cutured for 6 h (**B**).

The damage of limonene on the cell membrane of *L. monocytogenes* was further explored by determining the content of intracellular soluble protein in cells, and SDS-PAGE verified the results [18]. As shown in Figure 6A, the protein concentration of the control group increased with the growth of bacteria during 0–9 h and then decreased at 12 h. The addition of limonene (1 MIC) caused a sharp decrease in the protein concentration of *L. monocytogenes* from 0–12 h (*p* < 0.05). The protein concentration of the control group was significantly higher than that of the treatment group (*p* < 0.05). As seen from the gel electrophoresis image (Figure 6B), the protein band corresponding to the limonene (1 MIC) treatment was reduced when compared with that corresponding to the control group.

#### 2.2.6. Effect of Limonene on ATP concentration and ATPase

**Figure 7:**
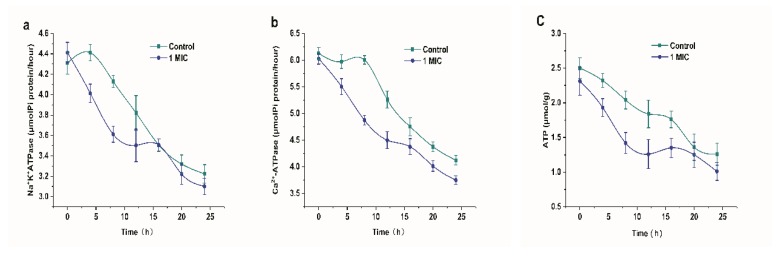
Changes in Na^+^-K^+^-ATPase (**a**) and Ca^2+^-ATPase (**b**) activities of *L. monocytogenes*. The effect of Limonene on ATP concentration of *L. monocytogenes* (**c**).

Figure 7 shows the effect of limonene on the ATP concentration and ATPase of *L. monocytogenes*. The addition of limonene resulted in a decrease in Na^+^K^+^-ATPase and Ca^2+^-ATPase over 24 h, as shown in Figure 7a,b. The Na^+^K^+^-ATPase activity was extremely reduced in the 0–8 h group (*p* < 0.01) and was significantly lower in the 4–12 h group than in the control group (*p* < 0.05). In addition, the Ca^2+^-ATPase activity was extremely reduced in the 0–12 h group and it was significantly lower in the 4–24 h group than in the control group (*p* < 0.05). Simultaneously, in Figure 7c, we observed that the ATP content showed a downward trend. At 8–16 h, the ATP content of *L. monocytogenes* that was exposed to limonene (1 MIC) was significantly decreased (*p* < 0.05). 

#### 2.2.7. Effect of Limonene on Respiratory Chain Complex I~V of *L. monocytogenes*

**Figure 8:**
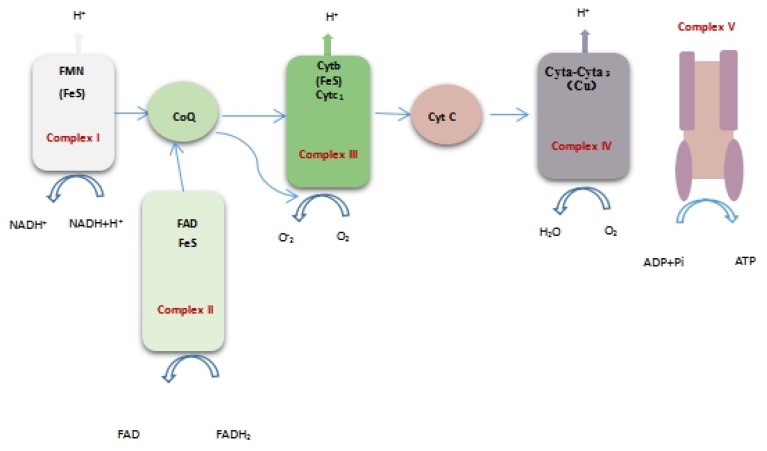
Oxidative phosphorylation system.

**Figure 9:**
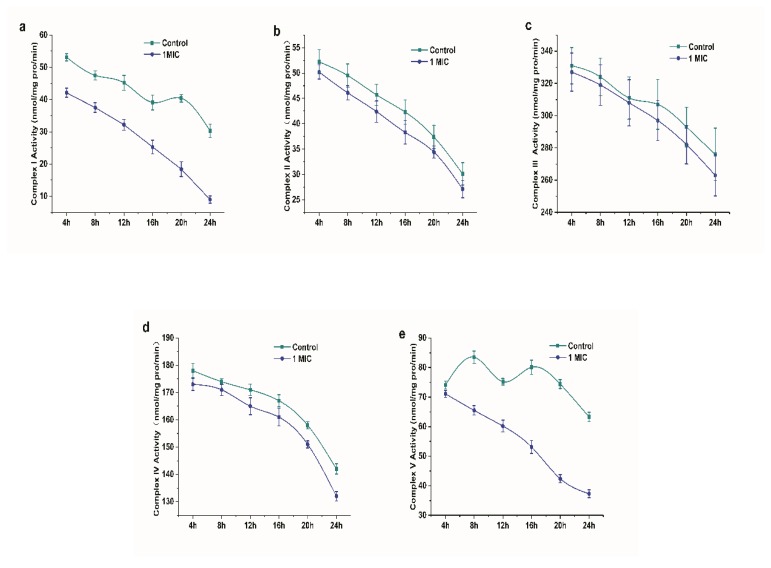
Changes in the activity of respiratory chain complex I~V of *L. monocytogenes*. (**a**) complex I, (**b**) complex II, (**c**) complex III, (**d**) complex IV, and (**e**) complex V.

Respiratory chain enzyme complexes are proteins that are related to oxidative phosphorylation. Complex I (NADH-ubiquinone oxidoreductase), complex II (succinate dehydrogenase), complex III (ubiquinol-cytochrome c reductase), complex IV (cytochrome oxidase complex), and complex V (ATP synthase) are mainly included as the respiratory chain complexes. The respiratory function can be directly or indirectly reflected by the activity of respiratory chain complexes [19]. Figure 8 shows the position of the respiratory chain complex in the respiratory chain. Figure 9 shows the changes in the I~V activity of the *L. monocytogenes* respiratory chain complex with time. The activity of complexes I~V of the *L. monocytogenes* respiratory chain showed a downward trend. In addition, the activity of respiratory chain complex I and complex V treated with limonene at 8–24 h was significantly lower than that of the control group (*p* < 0.05). 

#### 2.2.8. Effect of Limonene on Differential Protein Expression of Respiratory Chain Complex of *L. monocytogenes*

Table 2 shows the effects of limonene on the expression of respiratory chain-related complexes. The results indicated that the protein units of complexes I and IV were slightly downregulated in different proteins that were treated with limonene for 0 h. The protein units of complexes I, III, IV, and V were significantly downregulated at 8 h. Among the differentially expressed proteins that were treated with limonene for 24 h, complexes III, IV, and V were significantly downregulated, and some of the protein units in complexes I and II were downregulated. It can be seen that the damage of limonene to the respiratory chain of bacteria showed an increasing trend with the prolongation of treatment time.

## 3. Discussion

This study explored the antibacterial activity and its mechanism of limonene against *L. monocytogenes*. Anis Ben Hsouna et al. [14] found that limonene was a main component in C. limon essential oil (ClEO) by GC-MS, and the antibacterial activity of C. limon essential oil was assessed by evaluating the inhibition zone (IZ) and determining the MIC values. Among Gram-positive bacteria, the highest inhibitory zone was observed against *L. monocytogenes* (26 mm), and the MIC value was 0.039 ± 0.3 mg/mL. In this study, the results of MIC and the growth curves of *L. monocytogenes* indicated that limonene had a significant inhibitory activity and it could effectively inhibit the growth and reproduction of *L. monocytogenes*. However, the MIC of Levofloxacin Hydrochloride (0.625 mL/L) was higher than the MIC of limonene (20 mL/L), which indicated that limonene was slightly less susceptible than Levofloxacin Hydrochloride against *L. monocytogenes*. The efflux pump is a kind of membrane transport protein, which is widely existed in *L. monocytogenes*, and can discharge antibiotics and other antimicrobial compound out of the cells to adapt to changes in the environment, so that the drug concentration in the bacteria is always kept at a low level, thus producing drug resistance. It can be speculated that *L. monocytogenes* may be tolerant to limonene [20]. 

The SEM images clearly showed that limonene could destroy the normal morphology of *L. monocytogenes*. The destruction of the cell wall and cell membrane could further lead to the leakage of some intracellular substances, such as extravasation of protoplasms, thus leading to cell death. A similar report revealed that the cell structure of Pseudomonas aeruginosa treated with limonene changed significantly, and the cells appeared to be sticky, swelled, and lysed, which was similar to the results of this experiment [21].

The cell membrane is an important protective barrier of bacteria [22]. When bacteria encounter strong bacteriostatic agents, the cell membranes of bacteria are destroyed, which causes the internal electrolyte to leak into the culture medium, and the conductivity of the culture medium then increases [23]. In this study, the conductivity was observed to significantly increase, which indicated that limonene could result in leakage of bacterial contents and change the permeability of the cell membrane, increasing the conductivity. When the cell membrane is intact, PI is blocked from binding to nucleic acids in the cell. PI can enter the cell and combine with nucleic acids, exhibiting red fluorescence, once the cell membrane is destroyed. Yan, F et al. [24] revealed that AACS can reduce cell viability by causing cell membrane rupture or increased permeability, thereby facilitating PI entry into cells and binding to nucleic acids. They also believed that the fluorescence was related to the concentration of AACS. The PI fluorescence staining results indicated that limonene treatment caused damage to the *L. monocytogenes* cell membrane and, as the concentration of limonene increased, the degree of damage to the cell membrane increased, eventually leading to cell death. The results were consistent with the results of the conductivity measurements.

We found an obvious increase in the optical density at 260 nm of the culture that was treated with limonene (1 MIC and 2 MIC), which showed that nucleic acids of *L. monocytogenes* were leaked out of the cell after treatment of limonene. Simultaneously, the protein concentration in *L. monocytogenes* was noticeably decreased after the addition of limonene (1 MIC). In addition, protein bands that were treated with limonene were lighter than that of the control group, which indicated that limonene could lead to the proteins leakage through cell membranes and play an antibacterial effect by affecting protein expression. A similar report revealed that ε-poly-lysine resulted in cell leakage, which was associated with the loss of intracellular materials (DNA, protein) [15]. Our experiments demonstrated that limonene treatment caused significant and irreversible damage to the bacterial structure and cell membrane permeability, thus resulting in the leakage of nucleic acids and proteins from cells.

ATPase plays a major role in that regulation of ion balance inside and outside mitochondrial membranes and energy metabolism [25,26]. Among them, Na^+^-K^+^-ATPase is crucial in the transmembrane transport of sodium and potassium ions and maintaining the permeability of the cell membranes [27]. Ca^2+^-ATPase can catalyse the hydrolysis of ATP on the inner side of the plasma membrane, which is significant in releasing energy and maintaining a low concentration of free Ca^2+^ in the cells [15]. Reference [28] reported that a rapid decrease in Na^+^-K^+^-ATPase activity could result in a difference in the H^+^ gradient inside and outside the cell membrane, thereby altering the permeability of the cell membrane. In addition, the decrease in Ca^2+^-Mg^2+^-ATPase activity will inevitably affect the function of the Ca^2+^ pump, break the balance of Ca^2+^ in cells, and even lead to intracellular Ca^2+^ overload, thus causing cell apoptosis [29]. ATP is the most basic carrier of energy conversion in living organisms, and the change in its content is directly related to the energy metabolism of the cells. This result indicated that the ATP concentration of *L. monocytogenes* exposed to limonene was significantly decreased, which could be due to the decrease in intracellular ATPase activity. Based on the above results, it can be inferred that limonene could lower the enzyme activity, inhibit the respiration, and break the ATP balance of *L. monocytogenes*. In addition, the permeability of the cell membrane was increased, so the energy and key substances that are required for the growth and reproduction of the cell cannot be synthesized in time, which ultimately leads to cell death.

The respiratory chain of *L. monocytogenes* located on the plasma membrane is a system of electrons that transfers electrons from NADH or FADH_2_ to oxygen, being accompanied by the production of O_2_ and ATP. Lorena Tapia et al. [30] analysed the changes in enzymes related to respiratory chain complexes after 13-EPI-sclareol treatment. Among them, NADH oxidase and cytochrome C reductase activities decreased, while the activities of coenzyme Q reductase and cytochrome C oxidase were not affected. These results indicated that the target site of 13-EPI-perillanol resided between coenzyme Q and cytochrome C. It should be noted in this study that the activity of respiratory chain complexes of *L. monocytogenes* decreased to different degrees. We inferred that limonene might attenuate respiration by inhibiting the activity of the respiratory chain complex, blocking the transmission of electrons from NADH to coenzyme Q, which might be an important cause of the blockage of ATP synthesis.

We found that the expression of the complex I subunit (CL1094.Contig4_CK_0A, CL1528.Contig4_CK_0A, CL4703.Contig1_CK_0A, Unigene11357_CK_0A), which is responsible for obtaining two electrons from NADH and transferring them to coenzyme Q via ferritin, was significantly upregulated [31], which indicated that more electrons would be transported from NADH into the respiratory chain of *L. monocytogenes* treated with limonene. Meanwhile, cytochrome (CL594.Contig2_CK_0A), one of the components of complex III, and cytochrome oxidase subunit (Unigene2340_CK_0A, Unigene7527_CK_0A, CL3277.Contig1_CK_0A), comprising the component of complex IV, were significantly downregulated, which indicated that the respiratory chain of *L. monocytogenes* treated with limonene was blocked and electrons accumulated in the respiratory chain. In addition, most of the ATP synthase subunits in complex V were significantly downregulated, indicating that ATP synthesis was blocked, which was consistent with the decrease in ATP content that is shown in Figure 7c. V-type proton ATPase subunit (Unigene6313_CK_0A) in complex V was significantly downregulated at 24 h. The V-type proton ATPase is an ATPase that relies on the energy produced by the hydrolysis of ATP to produce an electrochemical gradient across the membrane and it regulates the pH inside and outside the cell. The high expression of V-ATPase will form a microenvironment near the cells, and a high concentration of extracellular H^+^ will enter normal cells with a concentration gradient, which will cause necrosis and apoptosis of normal cells [32]. As shown in Figure 9, previous studies showed that limonene significantly inhibited the activity of respiratory chain complexes I ~ V of *L. monocytogenes*. The site of inhibition of bacterial respiration might exist in the process of NADH transfer to coenzyme Q in the electron transfer chain. According to the significant difference in protein expression, limonene induced tremendous changes in the expression of the respiratory chain enzyme complex proteins. The results showed that limonene could affect the respiratory function and the energy level of *L. monocytogenes* by inhibiting the proliferation of *L. monocytogenes* respiratory chain complex proteins, thus leading to bacterial death.

## 4. Materials and Methods

### 4.1. Bacterial Strains and Chemicals

Limonene ((+)-Limonene) was purchased from Tokyo Chemical Industry Co., Ltd. (Tokyo, Japan) and then emulsified in 20% ethanol. Levofloxacin Hydrochloride Eye drops was purchased from Guangdong Hongying Technology Co., Ltd. (Foushan, China). Tryptic Soy Broth (TSB) was acquired from Guangdong Huankai Microbial Sci. & Tech. Co., Ltd. (Guangzhou, China) (filtered twice and autoclaved at 121 °C before inoculation). PI was purchased from Jiangsu KeyGEN BioTECH Corp., Ltd. (Nanjing, China). The Bradford Protein Assay Kit was acquired from Beyotime Institute of Biotechnology (Shanghai, China). The Coomassie Brilliant Blue R-250 and Complex Activity Detection Kit were purchased from Solarbio Science & Technology Co., Ltd., (Beijing, China). Jiancheng Bioengineering Institute (Nanjing, China) provided the ATPase assay kit and the ATP assay kit. Guangdong Huankai Microbial Sci. & Tech. Co., Ltd. (Guangzhou, China) provided the strain *L. monocytogenes* FSCC 178006 (Lot number 10068B) and it was activated in TSB at 37 °C with shaking for 24 h. Limonene was of Specific grade. All other chemicals were of analytical grade.

### 4.2. Antibacterial Susceptibility and Determination of Bacterial Growth Curves

#### 4.2.1. Determination of Minimum Inhibitory Concentration (MIC)

The agar dilution method was applied to examine the antimicrobial susceptibility of limonene against *L. monocytogenes* [33]. Limonene and Levofloxacin Hydrochloride (dissolved in 20% ethanol, *v*/*v*, shaken in an oscillator for 2 min.) were incubated with *L. monocytogenes* (10^6^~10^7^ CFU mL^−1^) on tryptic soy agar plates at a final concentrations of 40, 20, 10, 5, 2.5, 1.25, 0.625, or 0 mL/L for 24 h at 37 °C. Each group (limonene-treated and untreated) included three biological replicates. The groups with sterile water and ethanol were marked as blank control and negative control, respectively. The groups with Levofloxacin Hydrochloride were marked as a positive control. The minimum dilution concentration for invisible bacterial growth was defined as the MIC [34].

#### 4.2.2. Determination of Bacterial Growth Curves of *L. monocytogenes*

The growth curve of *L. monocytogenes* was tested by the ultraviolet spectrophotometry method [35]. First, the bacterial solution was cultured to logarithmic growth phase (approximately 10^6^~10^7^ CFU/mL) and then inoculated in TSB medium at a 2% dosage. Afterwards, limonene was added to the TSB medium at final concentrations of 1 MIC and 2 MIC. Sterile water and ethanol (20%, *v*/*v*) were used as controls. Subsequently, the bacterial suspension was incubated at 37 °C and shaken at 150 rpm. Finally, the absorbance at 600 nm was determined by an ultraviolet spectrophotometer (TU1810, Beijing Purkinje General Instrument Co., Ltd., Beijing, China) every 1 h. 

### 4.3. Antibacterial Mechanism

#### 4.3.1. Scanning Electron Microscope (SEM) Analysis

The influence of limonene on the cell morphology of *L. monocytogenes* was examined by SEM (S-4800, Hitachi, Tokyo, Japan) [36]. The *L. monocytogenes* cells in the logarithmic growth phase were incubated with limonene (1 MIC and 2 MIC) for 6 h and 12 h at 37 °C in TSB. Sterile water and ethanol were used as controls. Afterwards, the suspension was washed and centrifuged (6000 rpm, 10 min.) with phosphate buffer solution (0.1 M, PBS, pH 7.2) three times. The cells were sequentially dehydrated with 20%, 40%, 60%, 80%, and 100% ethanol solution. Subsequently, the cells were collected and precooled for 2 h at −20 °C. Subsequently, the cells were dried for 12 h by a freeze dryer (Han Mei Ecology Instrument Co., Ltd., Beijing, China). Finally, the samples were sputter-coated with gold under vacuum and then observed.

#### 4.3.2. Effect of Limonene on Cell Membrane Permeability of *L. monocytogenes*

The influence of limonene on the membrane permeability of *L. monocytogenes* was examined by measuring the conductivity [37]. Limonene (1 MIC and 2 MIC) was added to the bacterial solution at logarithmic phase and then cultured at 37 °C. Sterile water was marked as a control. The bacterial suspension (5 mL, collected at 0, 2, 4, 6, 8, 10 h) was centrifuged at 6000 rpm for 10 min. Finally, the supernatant was diluted five times, and the conductivity was determined by a conductivity meter (Mettler Toledo, Switzerland).

#### 4.3.3. Determination of Cell Death of *L. monocytogenes*

Propidium iodide staining and inverted fluorescence microscopy (MOTIC CHINA GROUP CO., LTD., Fujian, Xiamen, China) were used to identify cell death [24]. The cells that were grown to the logarithmic growth phase were cultured with 1 MIC limonene (1 MIC and 2 MIC) for 6 h at 37 °C and then centrifuged at 2000 rpm for 5 min. at 4 °C. Afterwards, the cells were washed three times and then resuspended in 1× buffer. Subsequently, the initial concentration was adjusted to 1 × 10^6^ CFU/mL. Subsequently, 95 μL of the bacterial suspension was supplemented with 5 μL of PI at room temperature for 5 min. Sterile water was used as a control. Finally, 10 μL of the bacterial suspension was observed and then photographed under an inverted fluorescence microscope (excitation wavelength 536 nm, emission wavelength 617 nm).

#### 4.3.4. Nucleic Acid Leakage of *L. monocytogenes*

The bacterial suspension was separated into several flasks. Except for the control, *L. monocytogenes* cells that were grown to the logarithmic growth phase were treated with limonene (1 MIC and 2 MIC) and then cultivated at 37 °C in a constant temperature incubator shaker (Ao Hua Instrument Co., Ltd., Changzhou, China). Sterile water was used as a control. The bacterial suspension (5 mL, collected at 3 h and 9 h) was centrifuged at 6000 rpm for 10 min. The precipitation was discarded, and the supernatant of 3 mL was removed from the centrifugal tubes. Finally, a microplate reader determined the absorbance of DNA and RNA macromolecule substances in the supernatant (Flash Spectrum Biological Technology Co., Ltd., Shanghai, China) at 260 nm [18]. 

#### 4.3.5. Determination of Protein Concentration and Leakage of Protein

##### Determination of Protein Concentration

Cells that were grown to the logarithmic growth phase were treated with limonene (1 MIC) to determine changes in the protein concentration after limonene treatment, and then collected after incubating for 0, 3, 6, 9, and 12 h at 37 °C. Sterile water was used as a control. The bacterial suspension (5 mL) was centrifuged (6000 rpm, 10 min., 4 °C), and the precipitate was washed three times with PBS (0.1 M, pH 7.4). The prepared bacterial cells were resuspended in 5 mL of PBS, and the cells were lysed by lysozyme (0.1 mg/mL) for 30 min. Subsequently, the bacterial cells were further broken by ultrasonic treatment (power 300 W, ultrasound 4 s, interval 5 s) for 10 min. in an ice bath. Subsequently, the bacterial cells were centrifuged, and the soluble proteins in the supernatant were collected. Finally, Bradford by a microplate reader quantified the protein contents of the samples (Flash Spectrum Biological Technology Co., Ltd., Shanghai, China) [19,38].

##### Leakage of Proteins

The protein leakage of *L. monocytogenes* treated with limonene for 6 h was tested by sodium dodecyl sulfate-polyacrylamide gel electrophoresis (SDS-PAGE). The specific methods were as follows. First, the soluble protein in the supernatant treated with limonene (1 MIC) for 6 h was collected. Sterile water was marked as a control, and the amount of protein leakage from *L. monocytogenes* cells was then tested while using SDS-PAGE. Twenty microlitres of the prepared 5 × loading buffer was mixed with 5 μL of the protein sample in tubes, and the protein was denatured in boiling water for 5 min; then, 10 μL of the sample was loaded. Moreover, the concentrations of the separating gel and concentrating gel were 15% and 5%, respectively. The starting voltage was 60 V and the voltage of the sample changed to 120 V after entering the separating gel. After completing the electrophoresis, the gel was removed and then stained with Coomassie Brilliant Blue R-250, and the separated protein bands were obtained after decolorization [39].

#### 4.3.6. Effect of Limonene on the ATP

##### Effects of Limonene on the Activity of ATPase

Changes in the ATPase activity of *L. monocytogenes* were tested while using an ATPase assay kit and the absorbance values of the extract at 660 nm were determined by a UV spectrophotometer (Metash Instruments Co., Ltd., Shanghai, China). The enzyme activity unit was defined as the amount of inorganic phosphorus produced by ATP enzymatic decomposition of ATP per milligram of protein per hour.

##### Determination of ATP Concentration

Limonene at a final concentration of 1 MIC was added to the prepared bacterial suspension and then cultured at 37 °C for 0, 4, 8, 12, 16, 20, and 24 h, and then centrifuged at 6000 rpm for 10 min. to collect the bacteria precipitate. Sterile water was used as a control. The acquired bacteria precipitate was treated, as previously described in 4.3.5.1, to obtain the bacterial lysate. Finally, the intracellular ATP concentration was tested while using the ATP assay kit and then determined by a microplate reader (Flash Spectrum Biological Technilogy Co., Ltd., Shanghai, China) at 636 nm [40].

#### 4.3.7. Effects of Limonene on Respiratory Chain Complex I~V

The Complex Activity Detection Kit was used to determine the effect of limonene on the respiratory chain complexes I~V of *L. monocytogenes*. The specific steps were, as follows: first, 5 × 10^6^ bacterial cells of the control group (sterile water) and the experimental group (1 MIC) cultured for 4, 8, 12, 16, 20, and 24 h were collected; then, 1 mL of lysis buffer was added, and the cells were homogenized on ice with a homogenizer. The homogenate was centrifuged (4 °C, 600 g, 10 min.), and the supernatant was placed into another centrifugal tube and then centrifuged by centrifugation (4 °C, 11000 g, 15 min.). Next, 400 µL of lysis buffer was added to the precipitate, and the cells were broken by ultrasonic treatment (power 20%, ultrasonic 5 s, interval 10 s, repetition 15 times). The OD340, OD605, OD550, OD550, and OD660 were measured by a UV spectrophotometer to determine the enzyme activity of the respiratory chain complexes (I~V), respectively.

#### 4.3.8. Effects of Limonene on the Differential Protein Expression of the Respiratory Chain Complex of *L. monocytogenes*

The respiratory chain complex proteins were extracted according to 4.3.7 references. Protein samples were reduced and alkylated with iodoacetamide (carbamidomethylated), digested with trypsin, and labelled with iTRAQ reagent. Subsequently, SCX chromatography separated the labelled peptides (GE Healthcare). LC-ESI-MS/MS identified the resulting peptides. Protein function descriptions were described according to the NCBI and UniProt_Swissprot databases. Differentially using expressed proteins were classified the following scale: more than 1.2-fold, and the P value was less than 0.05.

### 4.4. Statistical Analysis

The experimental results were statistically analysed by SPSS software (version; IBM Corp., Armonk, NY, USA). The experiments were carried out in triplicate and expressed as the mean ± SD. The significant differences were determined at a significance level of *p* < 0.05. Graphs were created by Origin software (Origin Lab Co., Pro.17.0, Northampton, MA, USA).

## 5. Conclusions

The current work confirmed that limonene showed effective antibacterial activity against *L. monocytogenes*, but it was less susceptible than the positive control. Besides, the results showed that limonene could inhibit the growth of bacteria and even lead to death. In addition, limonene could destroy the cell wall and cell membrane, which leads to the leakage of proteins and nucleic acids. Simultaneously, limonene could inhibit ATP synthesis by inhibiting ATPase activity and respiratory chain complex activity, leading to respiratory metabolic disorders and, ultimately, cell death. In conclusion, this study showed that limonene can act as a potential inhibitor against *L. monocytogenes*. We will continue to use more advanced experimental technology to strengthen the antibacterial activity and explore the antibacterial mechanism of limonene in the future research.

## Figures and Tables

**Table 1:** Minimum inhibitory concentration (MIC) of limonene against *L. monocytogenes.*

Treatment	Concentration of Drugs (mL/L)								
		0.3125	0.625	1.25	2.5	5	10	20	40
**Limonene**		+++	+++	+++	+++	+++	++	-	-
**Levofloxacin Hydrochloride**		+	-	-	-	-	-	-	-
**Sterile water**	+++								
**20% Ethanol**	+++								

“+++”, a large number of colonies; ++, more colonies; “+”, a medium number of colonies; ‘‘-’’, no colonies.

**Table 2:** Effects of Limonene on Differential Protein Expression of Respiratory Chain Complex of *L. monocytogenes.*

Accession ^a^	Description ^b^	Fold ^c^			mitochondria
		0 h	8 h	24 h	l Complexes ^d^
Unigene6313_CK_0A	V-type proton ATPase subunit	0.93	1.08	1.17	Complex V
Unigene2707_CK_0A	V-type proton ATPase subunit	0.86	0.54	0.51	Complex V
CL3974.Contig1_CK_0A	ATP synthase subunit	1,02	0.66	0.62	Complex V
CL2659.Contig1_CK_0A	ATP synthase subunit	1.07	0.99	0.72	Complex V
CL1630.Contig1_CK_0A	ATP synthase subunit	1.15	0.92	0.77	Complex V
CL1719.Contig1_CK_0A	V-type proton ATPase subunit	0.97	0.94	0.83	Complex V
CL3569.Contig2_CK_0A	ATP synthase complex subunit	1.01	0.62	0.29	Complex V
Unigene2720_CK_0A	ATP synthase subunit	1.09	0.92	0.82	Complex V
CL2386.Contig2_CK_0A	ATP synthase subunit	1.22	0.99	0.73	Complex V
Unigene2340_CK_0A	Probable cytochrome c oxidase subunit	0.98	0.71	0.65	Complex IV
Unigene7527_CK_0A	Cytochrome c oxidase subunit	1.27	0.89	0.67	Complex IV
CL3277.Contig1_CK_0A	Cytochrome c oxidase subunit	0.91	0.72	0.59	Complex IV
CL594.Contig2_CK_0A	Ubiquinol-cytochrome c reductase complex subunit	0.99	0.59	0.48	Complex III
CL3198.Contig1_CK_0A	Succinate dehydrogenase iron-sulfur subunit	1.05	0.82	0.62	Complex II
CL1338.Contig1_CK_0A	Succinate dehydrogenase cytochrome B subunit	0.96	1.09	1.62	Complex II
CL3139.Contig2_CK_0A	PX domain-containing protein	1.22	1.26	1.37	Complex II
Unigene3114_CK_0A	NADH-ubiquinone oxidoreductase 9.5 k Da subunit	0.99	0.59	0.48	Complex I
CL3590.Contig2_CK_0A	Lactobacillus shifted protein	0.97	0.83	0.71	Complex I
Unigene4104_CK_0A	NADH-ubiquinone oxidoreductase subunit	1.03	0.87	0.63	Complex I
Unigene11357_CK_0A	NADH-ubiquinone oxidoreductase subunit	0.97	0.65	0.79	Complex I
Unigene13799_CK_0A	NADH-ubiquinone oxidoreductase subunit	1.03	1.18	0.72	Complex I
CL1094.Contig4_CK_0A	NADH-ubiquinone oxidoreductase subunit	0.89	1.05	1.33	Complex I
CL1528.Contig4_CK_0A	NADH-ubiquinone oxidoreductase subunit	0.99	1.17	1.42	Complex I
CL4703.Contig1_CK_0A	NADH dehydrogenase subcomplex	0.70	1.19	1.28	Complex I
Unigene11357_CK_0A	External alternative NADH-ubiquinone oxidoreductase	1.08	1.17	1.54	Complex I
CL3151.Contig2_CK_0A	NADH-ubiquinone oxidoreductase subunit	1.38	0.94	0.86	Complex I
Unigene4104_CK_0A	NADH-ubiquinone oxidoreductase subunit	1.08	0.96	0.79	Complex I
CL2913.Contig2_CK_0A	NADH-ubiquinone oxidoreductase subunit	0.89	0.81	0.69	Complex I

Accession ^a^ was identified by ESI MS/MS. Description ^b^ protein function description was referred to NCBI and Uniprot_Swissprot database. Fold ^c^ showed the protein expression and the ratio of the control group in the treatment group. Miochondrial Complexes ^d^ refers to which complex the protein belongs to. Red and green are used to label proteins with a difference of more than 1.2 times, green is down-regulated and red is up-regulated.
